# Atypical Scrapie Isolates Involve a Uniform Prion Species with a Complex Molecular Signature

**DOI:** 10.1371/journal.pone.0027510

**Published:** 2011-11-11

**Authors:** Dorothea R. Götte, Sylvie L. Benestad, Hubert Laude, Andreas Zurbriggen, Anna Oevermann, Torsten Seuberlich

**Affiliations:** 1 NeuroCentre, National and OIE Reference Laboratories for BSE and Scrapie, Division of Experimental Clinical Research, Vetsuisse Faculty, University of Berne, Berne, Switzerland; 2 Department of Pathology, Norwegian Veterinary Institute, Oslo, Norway; 3 3U892 Virologie Immunologie Moléculaires, Institut National de la Recherche Agronomique, Jouy-en-Josas, France; Creighton University, United States of America

## Abstract

The pathobiology of atypical scrapie, a prion disease affecting sheep and goats, is still poorly understood. In a previous study, we demonstrated that atypical scrapie affecting small ruminants in Switzerland differs in the neuroanatomical distribution of the pathological prion protein (PrP^d^). To investigate whether these differences depend on host-related vs. pathogen-related factors, we transmitted atypical scrapie to transgenic mice over-expressing the ovine prion protein (tg338). The clinical, neuropathological, and molecular phenotype of tg338 mice is similar between mice carrying the Swiss atypical scrapie isolates and the Nor98, an atypical scrapie isolate from Norway. Together with published data, our results suggest that atypical scrapie is caused by a uniform type of prion, and that the observed phenotypic differences in small ruminants are likely host-dependant. Strikingly, by using a refined SDS-PAGE technique, we established that the prominent proteinase K-resistant prion protein fragment in atypical scrapie consists of two separate, unglycosylated peptides with molecular masses of roughly 5 and 8 kDa. These findings show similarities to those for other prion diseases in animals and humans, and lay the groundwork for future comparative research.

## Introduction

Transmissible spongiform encephalopathies (TSEs), such as scrapie in sheep and goats, bovine spongiform encephalopathy (BSE) in cattle, and Creutzfeldt-Jakob disease (CJD) in humans, are fatal neurodegenerative diseases that are caused by prions, which are infectious misfolded proteins [1]. The neuropathology of the TSEs includes spongiform vacuolation, gliosis, and the aggregation of a pathological isoform, PrP^d^, of the endogenous host prion protein, PrP^c^, in the brain. According to the protein-only hypothesis, the PrP^d^ isoform is the infectious agent [2]. PrP^d^ differs biochemically from PrP^c^ in a number of its characteristics, which include the partial resistance to proteolytic degradation by proteinase K (PK). PK-resistant PrP^d^ fragments (PrP^res^) can be detected via immunochemical techniques such as Western blotting (WB) [3].

To date, three types of TSE have been found in small ruminants: classical scrapie, BSE, and atypical scrapie. Classical scrapie has been observed for more than two centuries [4]. Classical scrapie prions are transmitted between animals and via contamination of the environment, and may cause significant losses in affected small ruminant flocks. Experimentally, sheep and goats are susceptible to oral infection with BSE prions [5] and, recently, two goats have been described that were likely to have been naturally infected in the course of a BSE epidemic in cattle in Europe [6], [7]. For this reason, TSEs in small ruminants have been intensively monitored in European Union member states, however, to date, no further small ruminant BSE cases have been identified. Atypical scrapie was first observed in Norway in 1998 (hence, Nor98) and was later detected in a number of other countries primarily by means of active disease surveillance schemes [8], [9]. Such cases revealed discordant phenotypic features, in particular, SDS-PAGE PrP^res^ banding patterns and neuroanatomical PrP^d^ distributions that differ from those of classical scrapie and BSE. Moreover, these distinctions were frequently found in sheep that displayed prion protein genotypes associated with a relative resistance to classical scrapie, and there was rarely more than one animal per herd affected [10], [11].

Upon transmission into sheep and rodents models in the laboratory, this distinct phenotype was preserved, and it was concluded that atypical scrapie cases are caused by prion species different from those that underlie classical scrapie and BSE [12]–[16]. However, the pathobiology and phenotypic diversity of naturally occurring atypical scrapie remain to be elucidated. This lack of clarity results partly from the limited brain tissue available to researchers via active disease surveillance programs.

In a previous study, we had access to whole brains from small ruminants affected with atypical scrapie, and discovered a marked diversity in the neuroanatomical distribution of the PrP^d^ ([17], table 1). Similar findings were later reported by other groups in both naturally-occurring cases and following experimental oral transmission of atypical scrapie isolates to sheep [16], [18]–[20]. However, it remains to be determined whether this diversity in PrP^d^ distribution is attributable to host factors, the involvement of particular prion types or a combination of both.

**Table 1:** Small ruminant isolates, attack rates, and survival times in tg338 mice.#

Inocula	Species	PrP^d^ deposition intensity¶ [Obx/Cbl/Cbr]	PRNP Genotype§	Attack rate[N_d_/N_i_]	Mean survival time ± SEM [days]
**Atypical scrapie**					
G1/RS	Goat	1/-/-	**A_136_L_141_H_154_Q_171_/ALHQ**	17/17	235±6.5
G2/FS	Goat	1/1/3	**ALHQ/ALHQ**	14/14	205±5.0
S2/RS	Sheep	1/3/-	ALRR/ALRR	12/12	188±4.8
S3/RS	Sheep	1/3/-	ALRR/ALRR	16/16	227±4.2
S4/RS	Sheep	1/-/-	**ALHQ/ALHQ**	2/2*	189/256
S5/FS	Sheep	1/1/3	ALRQ/**ALHQ**	16/16	216±6.0
S6/FS	Sheep	0/3/0	ALRR/ALRR	14/14	213±6.0
S7/CS	Sheep	1/3/3	**AFRQ/AFRQ**	14/14	215±5.2
Nor98	Sheep	1/3/3	**ALHQ/ALHQ**	16/16	223±3.9
**Classical scrapie**					
Classical scrapie	Sheep	3/2/3	V_136_R_154_Q_171_/A_136_R_154_Q_171_	16/16	304±17
**Negative**					
Sham-inoculated	Sheep		n.a.	0/18	567±22.4

# abbreviations: Obx, obex; cbl, cerebellum; Cbr, cerebrum; n.a. not available; N_i_, number of mice inoculated; N_d_, number of mice diseased

¶ as reported in our previous study [17]; -, structure missing; 1, mild; 2, moderate; 3, severe.

*for S4/RS fourteen out of sixteen inoculated mice died within two days post inoculation, most probably due to a high bacterial contamination of the sample.

§ polymorphisms of the *PRNP* alleles are shown for position 136, 141,154 and 171 for the atypical scrapie isolates and for positions 136, 154 and 171 for the classical scrapie control. Alleles associated with relative susceptibility with atypical scrapie are indicated in bold.

In the current report, we have addressed this question by transmitting a panel of eight small ruminant atypical scrapie isolates, with different neuroanatomical PrP^d^ distribution patterns, into mice overexpressing the ovine prion protein. This mouse model has been shown to be highly susceptible to both classical and atypical scrapie prion pathology. We compared survival times, pathology attack rates, patterns of lesions, PrP^d^ distribution in the brain, and biochemical PrP^res^ characteristics between these different groups and with mice infected with isolates of classical scrapie and atypical/Nor98 scrapie. Our findings support that atypical scrapie, despite the phenotypic variations evident in the natural hosts, is caused by a uniform species of prion. Our characterization of atypical scrapie isolates also reveals a previously unrecognized complexity of the molecular PrP^res^ phenotype.

## Results

### Clinical signs of disease, attack rates and survival times

**Figure 1:**
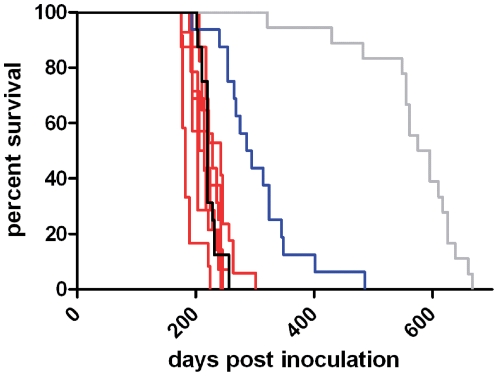
Survival of tg338 mice inoculated with different TSE isolates. Mice infected with Swiss atypical scrapie isolates (red) showed survival times similar to those infected with Nor98 (black), but these survival times were clearly shorter compared to the classical scrapie group (blue) and sham-inoculated mice (grey).

Following inoculation with atypical/Nor98 and classical scrapie, mice consistently showed characteristic clinical signs of TSE. At the terminal stage of the disease, the animals displayed ataxia and tremor, lost weight, and exhibited impaired grooming behavior and kyphosis. There were no obvious differences in clinical presentation between the isolates studied. As some of the sham-inoculated mice also showed neurological impairment at the end of their life-span, we defined mice as TSE positive or negative according to the presence or absence of PrP^d^ in the brain in either immunohistochemistry (IHC) or WB. All eight Swiss atypical scrapie isolates, two derived from goats and six from sheep, and the Nor98 isolate transmitted efficiently to tg338 mice following intracerebral inoculation, with attack rates of 100% (**Table 1**). The average survival times in the atypical scrapie groups including the Nor98 were very similar (between 188 and 235 days) and did not differ significantly between groups that were infected with isolates from donor animals of different *PRNP* genotypes. However, inoculation survival in all atypical scrapie isolates and Nor98 was significantly shorter (p<0.0001) compared to the classical scrapie isolate (304±17 days) and to the sham-inoculated mice (567±22 days **(** **Fig. 1** **)**).

### Histopathological brain lesion profiles

**Figure 2:**
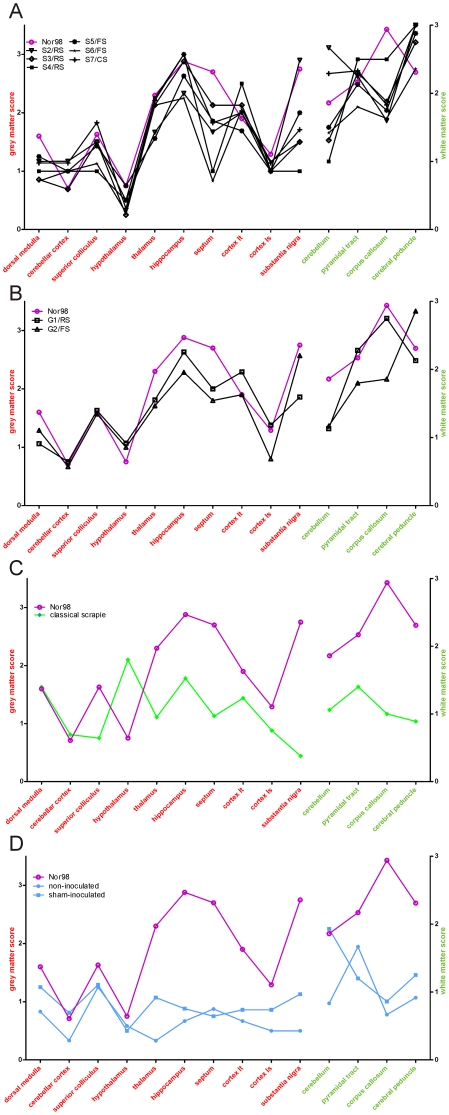
Lesion profiles in scrapie-infected tg338 mice. Lesions were scored according to the protocol established by Fraser and Dickinson [39] in ten grey matter areas (left y-axis, scale 0 to 5) and in four white matter areas (right y-axis, scale 0 to 3). All depicted values represent means of scores in mice inoculated with the same isolate: atypical sheep scrapie **(A)**, atypical goat scrapie **(B)**, classical scrapie **(C)** as well as sham-inculated and non-inoculated mice **(D)**. To facilitate cross-comparison, Nor98 scores are included in each graph.

**Figure 3:**
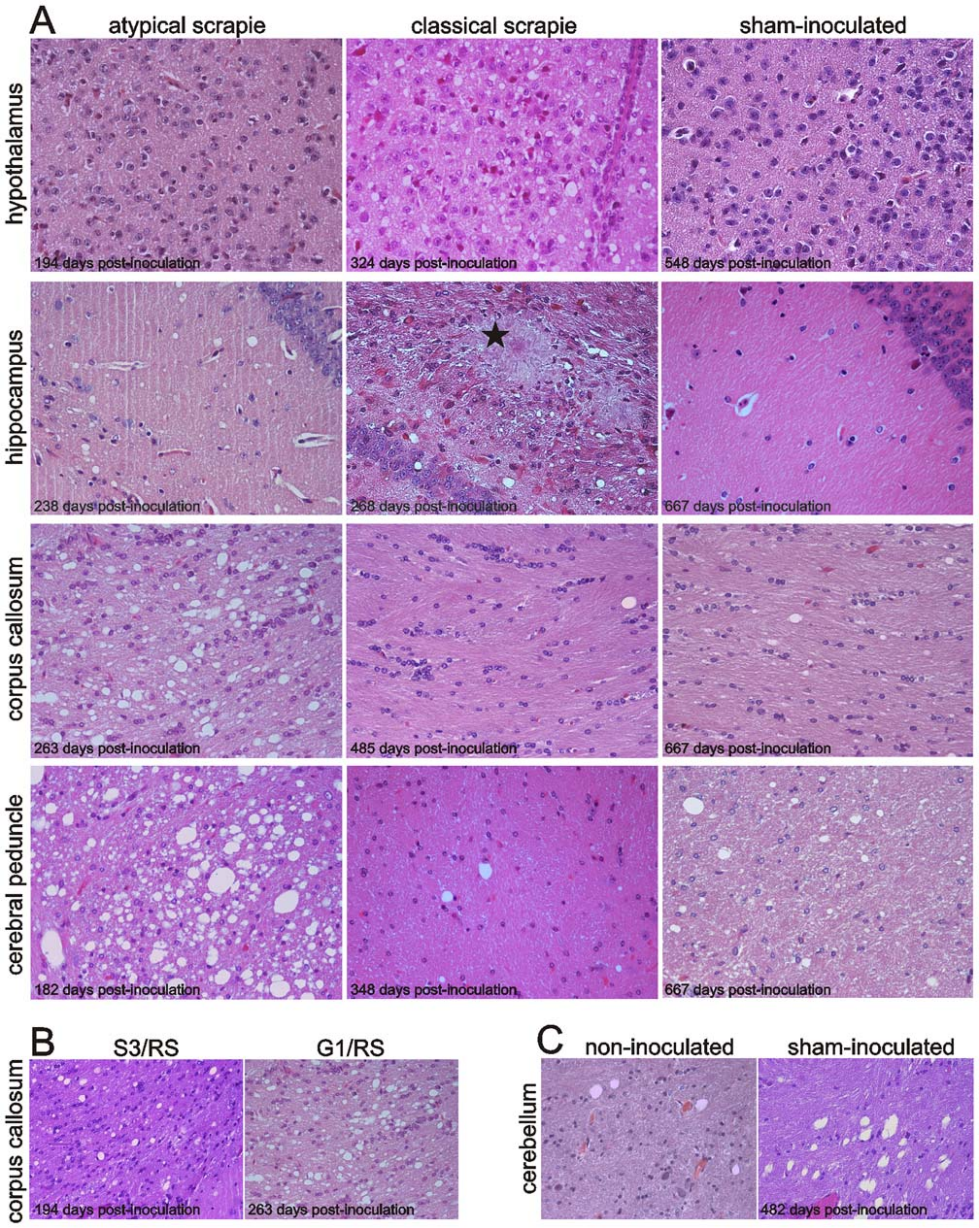
Representative spongiform pathology in tg338 mouse brains. **(A)** Vacuolar lesions are evident in the hypothalamus, hippocampus, corpus callosum, and cerebral peduncle of TSE-inoculated and control mice. Classical scrapie-inoculated mice exhibit plaques in various brain structures such as the hippocampus (star). **(B)** Variability of spongiform lesions in the corpus callosum between mice inoculated with atypical scrapie isolates. **(C)** Cerebellum of a non-inoculated mouse showing mild (left) and a sham-inoculated mouse showing severe (right) vacuolar lesions. All microphotographs are 40X magnifications of hematoxylin and eosin-stained mouse brain sections.

The intensity and neuroanatomical distribution of vacuolar lesions are very similar between mice infected with sheep (**Fig. 2A**) vs. goat (**Fig. 2B**) atypical scrapie isolates, and the Nor98 isolate. However, in the corpus callosum, a slight difference in the intensity of lesions is observed between mice infected with the Swiss atypical sheep isolates (**Fig. 3B**) and those with the Nor98 isolate (**Fig. 2A**). In addition, inoculation with the goat isolate G1/RS results in lesion scores similar to the Nor98 isolate (**Fig. 2B**), while the inoculation with the sheep isolate S4/RS results in intermediate lesion scores (**Fig. 2A**) in the corpus callosum. Vacuolar lesions were, in general, less severe following inoculation with the classical scrapie isolate, although the hypothalamus still exhibited considerable lesions in these mice (**Fig. 2C** **,** **Fig. 3A**). In both, sham- and non-inoculated mice, we observed mild vacuolar lesions in most of the brain structures investigated, and lesions were particularly notable in the white matter of the cerebellum (**Fig. 2D** **,** **Fig. 3C**).

### Neuroanatomical PrP^d^ distribution

**Figure 4:**
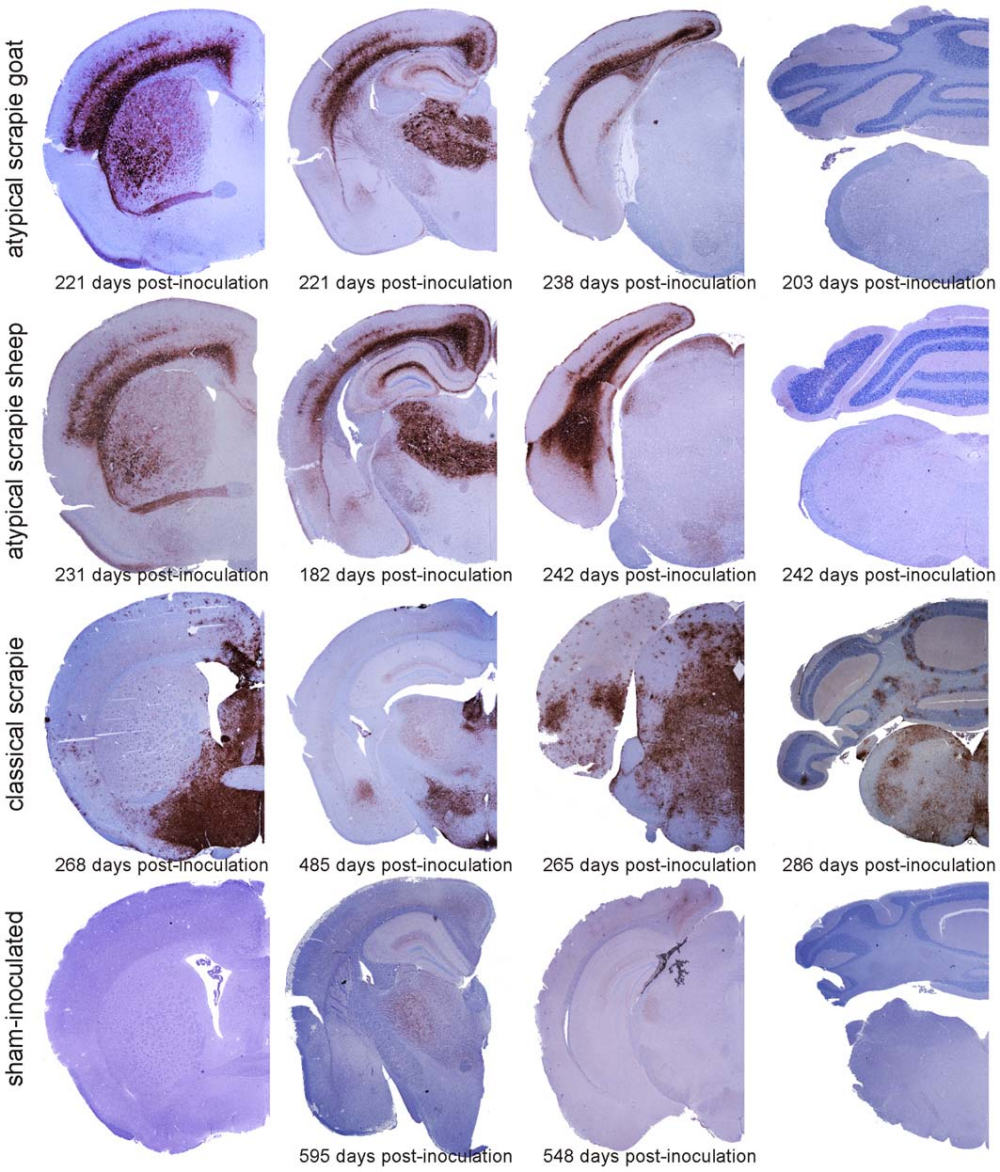
Neuroanatomical PrP^d^ distribution in mice inoculated with different scrapie isolates. Microphotographs of representative mouse brain sections stained for PrP^d^ by immunohistochemistry using antibody SAF84 are presented. For details on the anatomical structures, see **Fig S1**.

**Figure 5:**
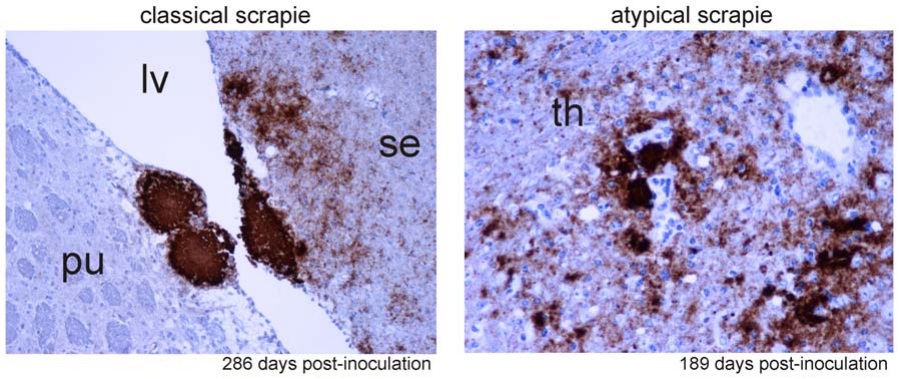
Localization and size of plaque-like PrP deposits in atypical and classical scrapie. In atypical scrapie-inoculated mice, relatively small plaques were observed in the neuropil (40X magnification), while the plaques in the classical scrapie-inoculated mice were larger, coalescing and always associated to the leptomeninges and the ependyma (20X magnification). PrP^d^ immunoreactivity was detected using the mAb SAF84. Neuroanatomical structures: septal nuclei (se), caudate putamen (pu), lateral ventricle (lv) and thalamus (th).

We assessed the distribution of the pathological PrP^d^ protein at four rostro-caudal positions in the brains of inoculated mice (**Fig. S1A**). The distribution pattern of PrP^d^ immunoreactivity is similar amongst the mice inoculated with the Swiss atypical scrapie isolates as well as the Nor98 isolate. However, the PrP^d^ distribution in these groups is strikingly distinct from that observed in the classical scrapie-inoculated mice (**Figs. 4** **, S1B**). The localization and size of plaque-like PrP^d^ deposits also differ greatly between the groups exposed to atypical vs. classical scrapie isolates (**Fig. 5**). These plaques are readily observable in hematoxylin and eosin-stained sections from mice inoculated to classical, but not atypical scrapie (**Fig. 3A**). In sham-inoculated mice, we observed a faint intraneuronal labeling in the cortex, the septal nuclei, and in some white matter structures, as well as a faint axonal staining in the thalamic nuclei (**Figs. 4** **, S1B**), the latter was also evident in non-inoculated tg338 mice (data not shown).

### Biochemical characteristics of PrP^res^ following transmission to tg338 mice

**Figure 6:**
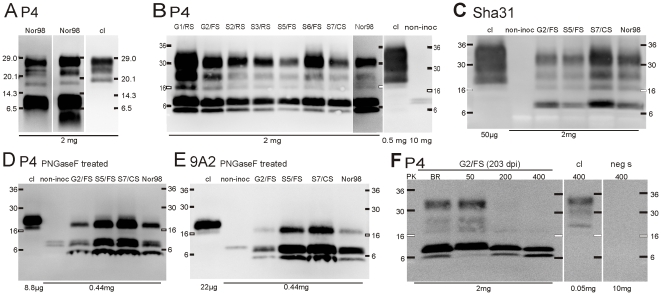
Molecular PrP^res^ typing of atypical scrapie affected tg338 mice. Western blots for PrP^res^ of representative brain extracts following SDS-PAGE using **(A)** 16.5% tris-glycine and **(B-F)** 4–20% tris-glycine gradient gels. Immunochemical detection was performed with antibodies P4, Sha31 or 9A2 as indicated. In some instances (**D**, **E)**, samples were deglycosylated with PNGaseF. **(F)** Atypical scrapie PrP^res^ banding pattern following sample pretreatment with different concentrations of proteinase K (PK, in µg/ml), compared to the PK digestion protocol of the Bio-Rad TeSeE confirmatory Western blot (BR). Identification codes for the atypical scrapie isolates are shown at the top. Controls: cl, classical scrapie affected tg338 mouse; non-inoc, non-inoculated tg 338 mouse; neg s, TSE negative sheep. Molecular mass standards in kDa and loaded tissue equivalents are indicated.

We used Western blotting to characterize the SDS-PAGE banding patterns of PrP^res^ using our standard monoclonal antibody P4-based protocol [17] and 16.5% polyacrylamide gels. As expected, the PrP^res^ WB patterns in the brains of the inoculated mice were similar with those previously published for classical and atypical scrapie, and consistent with the respective inocula. However, we identified an additional PrP^res^ fragment with a molecular mass <6.5 kDa in some immunoblots from mice inoculated with the atypical/Nor98 scrapie isolates (**Fig. 6A**). We then re-analyzed all the samples using 4-20% gradient tris-glycine polyacrylamide gels to better separate the low molecular mass proteins. These immunoblots were also probed with the P4 antibody and we found that all of the atypical scrapie samples, including the Nor98, showed the same pattern, with two distinct bands of lower molecular mass; one migrating at roughly 5 kDa, and another band at 8 kDa (**Figs. 6B** **and S2**). No bands were detected in the Western blot, when the P4 antibody was replaced by IgG purified from normal mouse serum (data not shown).

Sham-inoculated and non-inoculated control mice also showed some discrete PK-resistant bands in the Western blot. The bands were, in general, much less intense, visible only upon loading of relative large amounts of material and were not detected following more stringent PK-digestion (**Fig. S3**). However, in the majority of the sham-inoculated and non-inoculated mice we found an 8 kDa band of a relative high PK-resistance, which was reminiscent to that in the atypical scrapie infected mice (**Figs. 6 B-E** **and S3**).

Immunoblots containing representative atypical scrapie samples were then probed with the monoclonal Sha31 antibody, which is widely used in small ruminant TSE research and diagnostics and binds nearer to the C-terminus of the prion peptide than does P4 or the 9A2 antibody. After immunoblotting with Sha31, however, only the 8 kDa, but not the 5 kDa moiety of PrP^res^ could be detected (**Fig. 6C**). We then treated the protein lysates with the endoglycosidase PNGase F, which removes N-linked glycosylation. Treatment with PNGase F indicated that the fragments migrating at 5 kDa, 8 kDa, and 18 kDa correspond to unglycosylated PrP^res^ moieties, whereas those above 18 kDa are N-linked glycosylated. We found that immunoblotting with the 9A2 antibody resulted in the same pattern as P4, confirming the identity of these bands as PrP^res^ fragments (**Fig. 6D and E**). While the ∼18 kDa PrP^res^ fragment and its glycoforms were relatively sensitive to more stringent PK-digestion, this was not the case for the 5 and 8 kDa peptides (**Fig. 6F**).

### Biochemical PrP^res^ characterization of sheep/goat isolates

**Figure 7:**
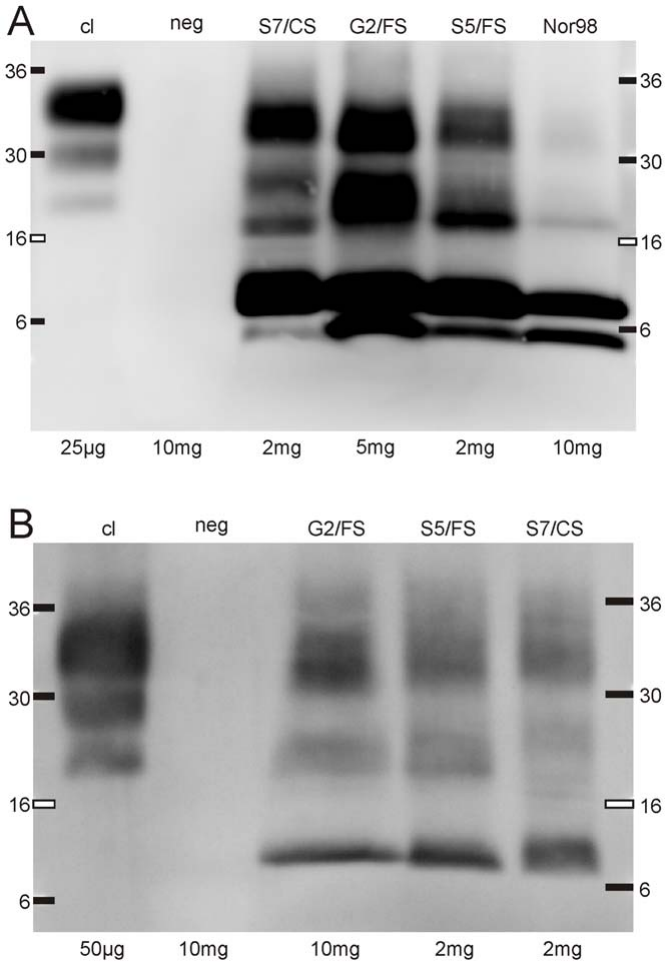
Molecular PrP^res^ typing of atypical scrapie-affected sheep and goats. SDS-PAGE was performed using 4–20% gradient gels and blots were probed with mAb P4 **(A)** and Sha31 **(B)**. Samples of a classical scrapie-affected sheep (cl) and a TSE-negative sheep (neg) served as controls. Molecular mass standards and tissue equivalents are indicated.

We then investigated whether the low molecular mass dual-banding-pattern observed in mice inoculated with atypical scrapie was relevant to atypical scrapie in small ruminants. To this end, we used 4–20% gradient SDS-PAGE to analyze protein lysates obtained from the sheep and goat tissue used to prepare the atypical scrapie inocula. Interestingly, we found that the banding patterns obtained from immunoblotting with P4 in the atypical sheep and goat scrapie samples greatly resemble those obtained from atypical scrapie-inoculated tg338 mice. Similar to the findings with the inoculated tg338 mice, the 8 kDa peptide, but not the 5 kDa peptide, was detected using Sha31 (**Fig. 7**). Noteworthy, neither the 8 kDa peptide nor other PK-resistant bands were observed in TSE negative sheep (**Figs. 7** **and S3B**). These results suggest that, in the natural host, both the 5 kDa and 8 kDa peptides are disease-specific, and that these fragments are C-terminally truncated to a different extent.

## Discussion

The current study seeks to delineate the host-related from the pathogen-related factors that affect the phenotype of atypical scrapie in small ruminants. We show that the transmission of a panel of defined atypical scrapie isolates from sheep and goats in Switzerland, which displayed distinct neuroanatomical PrP^d^ deposition patterns in the donor animals, resulted in a uniform disease phenotype in tg338 mice with respect to attack rates, survival times, PrP^d^ distribution, and the molecular pattern of the PrP^res^ peptides. Taken together, the overall phenotype of the Swiss atypical scrapie isolates was indistinguishable from that of the Nor98, but was clearly distinct from that of the classical scrapie isolate. Our data therefore corroborate previous findings that atypical scrapie involves a prion species distinct from those of classical scrapie. Moreover, our results indicate that the type of prion is identical in both the Nor98 isolate and the atypical scrapie cases presented in this work.

In our analysis of the severity and distribution of brain lesions, we observed some subtle differences between the various isolates. However, these differences fell within the range of the variability observed between individual mice within each isolate group, and are therefore not considered significant. An unexpected observation was that non-inoculated mice as well as sham-inoculated mice also displayed mild histopathological lesions and PrP immunoreactivity. In the latter, this was complemented by neurological impairment at advanced age (i.e., 570 days post-inoculation) in some animals. These findings could be due to aging, sanitary status of the mouse colony or the housing conditions. Also, we cannot completely rule out that the negative inoculum contained low-levels of prion infectivity. While this situation may hamper the interpretation of data when investigating prion isolates that reveal longer incubation periods, it does not impact on our ability to interpret the range of data produced with the atypical and classical scrapie isolates in the present study.

The phenotype of the Swiss atypical scrapie isolates in the tg338 model is strikingly similar to previously published data on atypical/Nor98 scrapie field isolates from Germany, Norway, France, and the UK that used both the tg338 mouse model as well as other ovine PrP transgenic mouse lines [21]–[23]. This suggests that there are no major differences between atypical scrapie prions isolated from various geographical origins and from sheep or goats. Arsac and collaborators [12] reported that atypical scrapie isolates from donor animals carrying *PRNP* alleles that were associated with a relative higher susceptibility to the disease (AF_141_RQ or AHQ) revealed a significantly shorter survival time compared to those derived from donor animals without such alleles in mice transgenic for the ovine ARQ *PRNP* allele. In our and others studies [13], [22] a similar effect for atypical scrapie isolates was not observed in tg338 mice, which may be related to higher PrP expression levels and/or more rapid evolution of the disease in this model. Our results thus provide indirect evidence that the observed variability in the neuroanatomical PrP^d^ distribution of atypical/Nor98 scrapie amongst small ruminants may result from, as yet unidentified, host factors rather than from the involvement of distinct prion agents. This situation contrasts sharply to that in classical scrapie, for which a large body of evidence points towards the existence of diverse prion strains, which differ in their phenotypic presentation in sheep and after transmission to mouse models [24]–[26]. It is conceivable that this strain diversity, as in other infectious diseases, results from serial transmission events and interactions with the host. In this respect, the classical scrapie isolate used in our study may not fully represent this class of TSE agents. However, the uniformity of the atypical scrapie isolates in the tg338 model, further questions the origin of this disease. Therefore, more data from transmission studies in small ruminants will be required to thoroughly characterize the etiology, pathogenesis and host-pathogen interaction in atypical scrapie.

The molecular PrP^res^ phenotypes in small ruminants and mice affected with atypical/Nor98 scrapie have been addressed in detail in a series of studies [12], [22], [27]–[30]. In these studies there is some disagreement regarding the estimated molecular mass of the prominent PrP^res^ band of lower molecular mass (∼7–12 kDa), which is most likely due to different electrophoretic conditions. However, this band is universally considered to represent a single truncated PrP^res^ fragment. Using gradient SDS-PAGE and a set of PrP-specific monoclonal antibodies, we now show that this band in fact involves two distinct PrP^res^ fragments of approximately 8 kDa and 5 kDa. This dual-banding-pattern has likely been overlooked in the past, since to our knowledge all studies that focused on the molecular PrP^res^ properties in atypical scrapie used 12-15% SDS PAGE, which do not resolve the 5 and 8 kDa peptides. Moreover, confirmatory Western blot techniques may miss the 5 kDa fragment, when relying on antibodies that bind to the center of the PrP^res^ (∼aminoacid 150), such as Sha31.

We detected the 8 kDa PrP^res^ peptide in atypical/Nor98 scrapie-affected tg338 mice, sheep, and goats using the P4, 9A2, and Sha31 antibodies, indicating that this fragment includes at least amino acids 93 to 155 of the full-length mature prion protein. This band was detectable with a much lower signal in negative control mice, and exhibited a high resistance to proteolysis. However, the 8 kDa PrP^res^ band was absent in TSE-negative sheep and clearly related to the atypical scrapie affected sheep and goats. This could be due to the over-expression of the ovine prion protein transgene in these mice, and may explain the relatively high susceptibility of these mice to atypical scrapie compared to other TSEs. In contrast, the 5 kDa PrP^res^ species was observed only in atypical scrapie-affected tg338 mice and small ruminants, but not in the negative control mice and healthy small ruminants. The 5 kDa peptide also did not react with the more C-terminal-directed Sha31 antibody, and thus differs from the 8 kDa PrP^res^ at its C-terminus.

Recently, we reported on a classical scrapie outbreak in a Greek goat flock, in which several animals exhibited a distinct C- and N-terminally ragged PK-resistant PrP^res^ fragment [31]. This peptide demonstrated the same antibody-binding properties, and was estimated to be at similar molecular mass as the 5 kDa PrP^res^ moiety described in the present study. At that time, it was unclear as to which type of prion disease this peptide was related. Our current findings support the idea that this fragment is related to atypical vs. classical scrapie, and that both prion species were present in the Greek flock. Future studies will conduct a direct comparison of these peptides by gradient SDS-PAGE, pending the receipt of material from mouse transmission studies of the Greek isolates.

PrP^res^ peptides of low molecular mass have also been described in other types of prion disease, such as Gerstmann-Sträussler-Scheinker disease [32], [33] and Creutzfeldt-Jakob disease [34], [35] in humans as well as in H-BSE in cattle [36], [37]. Forthcoming directions of research are likely to focus on more precise comparative analyses of truncated PrP^res^ peptides and their role in the biology of human and animal prion diseases.

## Materials and Methods

### TSE isolates

A total of eight atypical scrapie isolates from Switzerland [13], [17] were included in this study, all of which had been identified by active or passive surveillance for TSEs in small ruminants in the years 2004 and 2005. An atypical scrapie isolate from Norway (Nor98), a classical scrapie isolate from an experimentally-infected sheep (genotype donor sheep VRQ/VRQ; recipient VRQ/ARQ). A pool of medulla oblongata tissues from sheep that tested negative for TSEs by two independent TSE screening tests, and by immunohistochemistry (IHC) in different brain structures and lymphatic tissues, served as a sham-inoculum. Details on the isolates and controls are shown in **Table 1**.

### Transgenic mice and bioassays

Tg338 mice express the ovine VRQ PrP on a murine PrP knock-out background at 6- to 8-fold higher levels compared to sheep [13], [38]. Tg338 mice were imported from a specific-pathogen-free (SPF) holding facility and bred in-house in a biosafety level 3 and SPF environment. Groups of 16 to 20 tg338 mice per isolate (8 to 10 weeks of age) were inoculated intracerebrally (ic) with 20 µl of 10% [w/v] brain tissue homogenates under isoflurane anesthesia. Mice were monitored for clinical signs of TSE at least twice per week, and were sacrificed when clinical signs were observed that indicated end-stage TSE, or when a minimum survival time of 630 days had elapsed. At necropsy, the brain of every second animal was fixed in formalin for histopathology and immunohistochemistry. The remaining brains and the spleen and muscle tissues were snap-frozen in liquid nitrogen and stored at -20°C for western blot and ELISA analyses. Parallel samples were obtained from non-inoculated mice of variable age that had served for breeding purposes (n = 11). A mouse was considered to be TSE-positive when PrP^d^ was detected by Western blotting or immunohistochemistry in brain samples.

### Ethics statement

All animal experiments were conducted according to the “Ethical Principles and Guidelines for Experiments on Animals” by the Swiss Academy of Medical Sciences and were approved by the Committee for Animal Experiments of the Canton of Berne (license #10/08).

### Histopathology and lesion profiles

Mouse brains were fixed in 4% formaldehyde, decontaminated for 1 h in formic acid (98%) at room temperature, cut transversely into five pieces as suggested by Fraser and Dickinson [39], and embedded in paraffin. Four-µm thick tissue sections were stained with hematoxylin and eosin and vacuolar lesions were scored in ten defined grey matter areas on a scale from 0 to 5, and in four white matter areas on a scale from 0 to 3 [40].

### Immunohistochemistry for PrP^d^

The PrP^d^ immunohistochemistry was performed on the same brain structures used for the lesion profiling, with a NEXEX robot (Ventana instruments) and monoclonal SAF84 antibody (SPI bio, A03208, 1:100) as described elsewhere [41], at the Institute of Neuropathology, University of Zürich. The PrP^d^ deposition pattern was assessed at the NeuroCentre by microscopic examination of at least six mice per isolate, with the exception of isolate S4/RS, for which only one animal was available.

### Western blotting

Whole brains from mice, and brain samples of donor sheep and goats, were homogenized and purified using a commercial TSE test kit (Bio-Rad, 355–1165) according to the manufacturer's instructions, but with the following modification: the kit-provided PK of unknown concentration was replaced by PK (Roche, 03115828001) at a final concentration of 50 µg/ml, which corresponds to 250 µg PK per gram wet tissue. The resulting pellets were resuspended in Lämmli sample buffer (Bio-Rad, 161–0737). PrP^res^ fragments were separated by SDS-PAGE in hand-cast 16.5% tris-glycine gels or in pre-cast 4-20% gradient tris-glycine gels (Invitrogen, EC 6028), transferred to PVDF membranes (Bio-Rad, 162–0177), and blocked with 1% casein in PBS (Bio-Rad, 161–0783). Three PrP-specific mAbs were used: 9A2 (_102_WNK_104_)[42], P4 (_93_WGQGGSH_99_, r-biopharm, R8007), and Sha31 (_148_YEDRYYRE_155_, Bio-Rad TeSeE Western Blot, 355–1169). The epitopes on the prion protein recognized by these mAbs have been schematically presented in a previous study [31]. Immunoreactivity was detected using a polyclonal rabbit anti-mouse HRP antibody (Dako, P0260), a chemiluminescent detection system (ECL-plus Amersham Biosciences), and a LAS 3000™ imaging system (Fuji Film, Fuji Inc, Valhalla, NY). Protein bands were quantified and their molecular mass calculated with Quantity One software (Bio-Rad, Version 4.6.2) in comparison with a molecular mass standard (See-Blue, Invitrogen, LC5625).

### Deglycosylation treatment

Following the resuspension of precipitated protein pellets in TD4215 (4% [w/v] SDS, 2% [v/v] β-mercaptoethanol, 192 mM Tris, 5% [w/v] sucrose), proteins were deglycosylated by incubation with the endoglycosidase PNGase F for 90 min at 37°C (New England Biolabs, P0704L) according to the manufacturer's instructions. Samples were mixed with an equal amount of Lämmli sample buffer and processed for western blotting as described above.

### Statistical analyses

Survival time was defined as the number of days from inoculation to the animal's death. Animals that died in the first week following inoculation were excluded from the study. All statistical analyses were carried out using GraphPad's Prism software (Version 5.03). Survival times were compared between groups using the Log-rank Multiple-Comparison Test including the Bonferroni-correction (p<0.000758).

## Supporting Information

Figure S1 **Scheme of the neuroanatomical PrP^d^ distribution in groups of tg338 mice inoculated with different scrapie isolates**. **(A)** Presentation of the neuroanatomical structures investigated: cortex (cx), cingulum (cg), cerebral white matter (cw), corpus callosum (cc), caudate putamen (pu), septal nuclei (se), anterior commissure (ac), ventral pallidum (vp), piriform cortex (pi), hypothalamus (ht), lateral ventricle (lv), hippocampus (hi), hippocampal fissure (hf), habenula (ha), 3^rd^ ventricle (3v), thalamus (th), substantia nigra (sn), cerebral peduncle (cp), tractus opticus (ot), amygdala (am), superior colliculus (sc), nucleus brachium inferior colliculus (bi), pons (ps), aqueduct (aq), molecular layer (ml), granular layer (gl), cerebellar roof nuclei (rn), cerebellar white matter (wm), dorsal motor nucleus of the vagus nerve (dm), nucleus hypoglossus (hy), 4^th^ ventricle (4v), cerebellar peduncle (pe), spinal tract nucleus of trigeminal nerve (tn), spinal tract nucleus of trigeminal nerve (st), raphe (rf), pyramidal tract (pt). **(B)** PrP^d^ is depicted for mild (dots) and severe (filled areas) deposits in the grey (red) and white matter (green) structures separately. Amyloid PrP^d^ plaques were observed in the neuropil (★) as well as in apparent association with the cerebrospinal fluid space (⋆).
(TIF)

Figure S2 **Quantitative analysis of the PrP^res^ phenotype in atypical scrapie affected tg338 mice.** Molecular mass (y-axis, ±SD) and relative quantities of PrP^res^ bands (numbers [%] at the right side of each dot) are depicted as means from at least five mice per isolates following SDS-PAGE using 4–20% gradient gels and monoclonal antibody P4.
(TIF)

Figure S3 **Western blot banding patterns in the brains of sham-inoculated mice. (A)** Tg338 mice inoculated with TSE-negative sheep brain show variable banding patterns after treatment with PK (50 µg/ml). Survival times (in days) of individual mice are indicated on the top. The picture on the left shows an atypical scrapie affected mouse-brain sample (S7/CS) for comparison. **(B)**The brain sample of a sham-inoculated mouse (610 days post-inoculation [dpi]) was treated with different concentrations of PK (indicated on top in µg/ml; BR: PK of the Bio-Rad TeSeE Western Blot test kit of unknown concentration). Upper bands of both mice are fully digested when using higher PK concentrations whereas an 8 kDa band in sham-inoculated mice are relatively PK-resistant. Controls: classical scrapie (cl), negative sheep (neg s). Molecular mass markers are indicated at each side, tissue equivalents loaded per well at the bottom.
(TIF)
